# The effect of erythromycin and clarithromycin versus azithromycin on serum valproate concentration

**DOI:** 10.1016/j.jsps.2022.02.004

**Published:** 2022-02-09

**Authors:** Muradi Albanji, Samah Alshehri, Khalid Eljaaly

**Affiliations:** a Department of Pharmacy, King Abdulaziz University Hospital, Jeddah, Saudi Arabia; b Department of Pharmacy Practice, Faculty of Pharmacy, King Abdulaziz University, Jeddah, Saudi Arabia

**Keywords:** Interaction, Macrolide, Clarithromycin, Erythromycin, Azithromycin

## Abstract

**Introduction:**

Unlike azithromycin, erythromycin and clarithromycin strongly inhibit CYP450, which metabolizes valproic acid. The aim of this study was to evaluate the impact of macrolide administration on serum valproate trough levels.

**Methods:**

This retrospective cohort study included hospitalized adult patients who concomitantly received valproate with a macrolide. Patients who received a carbapenem, those who do not have a baseline and/or post-levels, and those who received different doses of valproate were excluded. The change in serum valproate trough level from baseline to after the occurrence of co-administration (post-level) was compared in patients who received either erythromycin or clarithromycin versus those who received azithromycin.

**Results:**

A total of thirteen patients were included in the comparison. The mean ± SD for change in serum valproate trough levels was significantly higher in the erythromycin/clarithromycin group than the azithromycin group (209.1 ± 105.9 µmol/L [equivalent to 30.1 ± 15.2 mg/L] vs. 12.7 ± 52.1 µmol/L [equivalent to 1.8 ± 7.5 mg/L]; P = 0.002).

**Conclusion:**

This study found a significantly higher increase in serum trough levels of valproate after co-administration of erythromycin/clarithromycin versus azithromycin. Clinicians should consider avoiding co-administration of erythromycin and clarithromycin with valproate if possible or close monitoring of valproate levels with dose reduction.

## 1 Introduction

Antiepileptic drugs (AEDs) are widely used in clinical practice and are associated with several clinically relevant drug-drug interactions (DDIs) (Johannessen Landmark and Patsalos, 2010). AEDs have a narrow therapeutic index, and modest pharmacokinetic alterations can result in loss of response or toxic effects (Johannessen Landmark and Patsalos, 2010). Third-generation AEDs have a better tolerability profile, fewer DDIs, and simpler pharmacokinetics when compared with older AEDs (Guerrini et al., 2012). However, antiepileptic management still relies on older AEDs as the main components, while third-generation AEDs are used only as add-on therapies (Shih et al., 2017). This complicates, rather than simplifies the DDI profile of AEDs.

AEDs are used for conditions other than epilepsy, such as bipolar disease, migraine, and chronic pain (Patsalos et al., 2002). Among old-generation AEDs, valproate (valproic acid) has become the most widely prescribed AED worldwide as it has various pharmacological effects via several mechanisms. Valproic acid is extensively metabolized by microsomal glucuronide conjugation, mitochondrial beta-oxidation, and cytochrome P450 (CYP450) isoenzymes (Levy et al., 2002). Valproate is a broad-spectrum AED that is effective against partial seizures (with or without secondary generalization) and/or primarily generalized tonic-clonic seizures and is regarded as a first-choice agent for most forms of idiopathic and symptomatic generalized epilepsies (Perucca, 2002).

AEDs are chronic medications used for long periods. Therefore, using co-administered medications for other conditions such as infections are likely and potential drug interactions are considerable (Patsalos et al., 2002). Valproic acid requires serum drug monitoring, and an optimal plasma concentration range of 350–700 µmol/L (50–100 mg/L) has been proposed (Patsalos et al., 2008). Some commonly used antimicrobials can inhibit valproate metabolism via CYP450 inhibition, such as certain macrolides (Eljaaly et al., 2019). Macrolide antibiotics are used for a variety of infections, particularly bacterial respiratory tract infections (Eljaaly et al., 2021, Eljaaly et al., 2017). Unlike azithromycin, erythromycin and clarithromycin strongly inhibit CYP450, but no previous studies to our knowledge evaluated the impact of administering macrolides on serum valproate levels. In one case report, a patient experienced symptoms and signs of valproate toxicity and an increase in valproate levels (260.4 µmol/L from a baseline of 88.8 µmol/L) after receiving erythromycin (Redington et al., 1992). This study aims to evaluate the impact of concomitant administration of different macrolides on serum valproate trough levels.

## 2 Methods

### 2.1 Study design and setting

This study was a single-center retrospective cohort study conducted at King Abdulaziz University Hospital, a large academic tertiary care hospital in Jeddah, Saudi Arabia. Ethical approval was obtained from the Unit of Biomedical Ethics Research Committee. Hospitalized adult (≥18 years) patients who concomitantly received valproate with a macrolide (erythromycin, clarithromycin, or azithromycin) between January 1, 2015 and December 31, 2019 were included. Patients who received a carbapenem (reduces valproate serum levels), those who did not have a baseline and/or post-levels during macrolide therapy, and those who received different valproate doses were excluded. Architect, an in vitro chemiluminescent microparticle immunoassay (CMIA), is used by our hospital for the quantitative measurement of valproate.

### 2.2 Data collection and study outcome

Data were obtained from the electronic database as well as the patients’ medical records using a standardized data collection tool. Baseline characteristics were collected, including the following: age, gender, weight, presence of renal or hepatic disease, interacting macrolide antibiotic, other interacting medications, antibiotic starting and stop dates, and the dose of affected medication (valproic acid), baseline trough level, post-level after starting the interacting macrolide. The primary outcome of the study was the change in serum valproic acid trough level from baseline to after the occurrence of co-administration (post-level).

### 2.3 Statistical analysis

Descriptive statistics were used to summarize the data. Means and standard deviations (SD) were used for normally distributed variables, while frequency counts and percentages were used for categorical variables. The difference between the two groups in mean change of serum valproate levels was compared using the unpaired *t*-test. We used SPSS for Windows version 23.0 (IBM Corporation, Armonk, NY, USA).

## 3 Results

Medical records of 286 patients who concomitantly received valproate with a macrolide during hospitalization were screened for eligibility. As a result, thirteen patients were included in the comparison (six patients in erythromycin/clarithromycin group and seven patients in azithromycin group). Out of 286 patients, 273 patients were excluded from the study for these reasons: 271 patients did not have both levels, one patient received a carbapenem before valproate post-level, and one patient received different doses of valproate.

The baseline characteristics of patients are summarized in Table 1. No significant differences were found between the two groups. The proportion of males was 54%. The mean age was 53 years in erythromycin/clarithromycin group and 51 years in azithromycin group. The mean dose of valproate was 557.1 mg in erythromycin/clarithromycin group and 575 mg in azithromycin group. None of the patients had renal disease, liver disease, or received concomitant medications known to interact with valproate.

**Table 1:** Patient baseline characteristics.

**Variables**	**Erythromycin/Clarithromycin (N = 6)**	**Azithromycin (N = 7)**
Age in years (Mean ± SD)	53 ± 9	51 ± 8
Male, n (%)	4 (66.7 %)	3 (42.8%)
Weight, kg (Mean ± SD)	77.3 ± 8.3	76 ± 8.6
Renal disease, n (%)	0	0
Liver disease, n (%)	0	0
Dose of Valproate, mg (Mean ± SD)	557.1 ± 191.7	575 ± 195.3

The mean ± SD duration from the time of starting macrolides until the post-level drawing time was 3.8 ± 1.3 days in the erythromycin/clarithromycin group and 3.3 ± 1.0 days in the azithromycin group. The range of change in serum valproate trough levels was between 83.7 µmol/L and 341.2 µmol/L in the erythromycin/clarithromycin group and between −65.9 µmol/L to 72.2 µmol/L in the azithromycin group. The mean ± SD change in serum valproate trough levels was significantly higher in the erythromycin/clarithromycin group than the azithromycin group (209.1 ± 105.9 µmol/L [equivalent to 30.1 ± 15.2 mg/L] vs. 12.7 ± 52.1 µmol/L [equivalent to 1.8 ± 7.5 mg/L]; P = 0.002).

## 4 Discussion

This study is the first to evaluate the drug-drug interaction between valproate and macrolides. A significantly higher increase in serum trough level of valproate was observed when it was co-administered with erythromycin/clarithromycin versus azithromycin. Guolden et al. reported that administering erythromycin in four children was associated with a mean increase of carbamazepine serum level by 21.8 µmol/L (equivalent to 3.1 mg/L) (Goulden et al., 1988). O’Connor and Fris observed increasing serum carbamazepine levels in five adults despite reducing the carbamazepine dose by 30–40% (O’Connor and Fris, 1994). These studies have small sample sizes, a limitation of our study as well. Future larger studies are needed to confirm our findings. It is worth mentioning that the majority of patients in our study were excluded due to inadequate monitoring of serum valproate levels which indicates a need for increasing awareness of ordering these levels with significant CYP450 inhibitors. Clinicians should consider replacing erythromycin and clarithromycin with azithromycin or other antibiotics depending on the indicated case. When valproate is used concomitantly with macrolides, monitoring valproate serum levels is required, and dose reduction of valproate might be needed. In addition, patients should be warned about the signs and symptoms of valproate toxicity.

## Declaration of Competing Interest

The authors declare that they have no known competing financial interests or personal relationships that could have appeared to influence the work reported in this paper.
